# Draft genome sequence data of a psychrophilic tundra soil methanotroph, *Methylobacter psychrophilus* Z-0021 (DSM 9914)

**DOI:** 10.1016/j.dib.2022.108689

**Published:** 2022-10-21

**Authors:** Antti Juhani Rissanen, Rahul Mangayil, Mette Marianne Svenning, Ramita Khanongnuch

**Affiliations:** a Faculty of Engineering and Natural Sciences, Tampere University, P.O. Box 541, FI-33014 Tampere, Finland; b Natural Resources Institute Finland, Latokartanonkaari 9, FI-00790 Helsinki, Finland; c Department of Bioproducts and Biosystems, School of Chemical Engineering, Aalto University, FI-00076 Aalto, Finland; d Department of Arctic and Marine Biology, UiT, The Arctic University of Norway, Tromsø 9037, Norway

**Keywords:** Methanotroph, *Methylobacter*, Psychrophilic, Cold ecosystem, Methane, Tundra, Boreal, Arctic

## Abstract

Psychrophilic methanotrophic bacteria are abundant and play an important role in methane removal in cold methanogenic environments, such as boreal and arctic terrestrial and aquatic ecosystems. They could be also applied in the bioconversion of biogas and natural gas into value-added products (e.g., chemicals and single-cell protein) in cold regions. Hence, isolation and genome sequencing of psychrophilic methanotrophic bacteria are needed to provide important data on their functional capabilities. However, psychrophilic methanotroph isolates and consequently their genome sequences are rare. Fortunately, Leibniz Institute, DSMZ-German Collection of Microorganisms and Cell Cultures GmbH was able to revive the long-extinct pure culture of a psychrophilic methanotrophic tundra soil isolate, *Methylobacter psychrophilus* Z-0021 (DSM 9914), from their stocks during 2022. Here, we describe the *de novo* assembled genome sequence of *Methylobacter psychrophilus* Z-0021 comprising a total of 4691082 bp in 156 contigs with a G+C content of 43.1% and 4074 coding sequences. The preliminary genome annotation analysis of Z-0021 identified genes encoding oxidation of methane, methanol and formaldehyde, assimilation of carbon and nitrate, and N_2_ fixation. In pairwise genome-to-genome comparisons with closely related methanotrophic strains, the strain Z-0021 had an average nucleotide identity (ANI) of 92.9% and 78.2% and a digital DNA-DNA hybridization (dDDH) value of 50.6% and 22% with a recently described psychrophilic, lake isolate, *Methylobacter* sp. S3L5C and a psychrotrophic, arctic wetland soil isolate, *Methylobacter tundripaludum* SV96, respectively. In addition, the respective similarities between genomes of the strains S3L5C and SV96 were 78.1% ANI and 21.8% dDDH. Comparison to widely used ANI and dDDH thresholds to delineate unique species (<95% ANI and <70% dDDH) suggests that *Methylobacter psychrophilus* Z-0021, *Methylobacter tundripaludum* SV96 and *Methylobacter* sp. S3L5C are different species. The draft genome of Z-0021 has been deposited at GenBank under the accession JAOEGU000000000.

**Specifications Table**

**Table:** 

Subject	Biological sciences
Specific subject area	Bacterial Genomics, Applied Microbiology and Biotechnology, Biogeochemistry, Environmental Microbiology
Type of data	Genomic sequence Table Figure
How the data were acquired	Whole genome sequencing of DNA extracted from type strain *Methylobacter psychrophilus* Z-0021 (DSM 9914) using Illumina Nextseq 550 Sequencing System.
Data format	Raw Assembled/Analyzed
Description of data collection	The DNA extraction, genome sequencing and genome assembly of type strain Z-0021 (DSM 9914) was a commercial service provided by Leibniz Institute, DSMZ-German Collection of Microorganisms and Cell Cultures GmbH. Genomic DNA was extracted, followed by library preparation. Sequencing was done using Illumina Nextseq 550 Sequencing System. Data was *de novo* assembled (SpaDES), annotated (Prokka, KofamKoala, PhyloPhlAn), and comparatively analyzed (Type Strain Genome Server, ANI Calculator).
Data source location	**Sequence data source** Institution: Tampere University City/Town/Region: Tampere Country: Finland **Type strain source** Leibniz Institute, DSMZ-German Collection of Microorganisms and Cell Cultures GmbH: https://www.dsmz.de/collection/catalogue/details/culture/DSM-9914
Data accessibility	Repository: The assembled whole-genome shotgun data (.fasta) has been deposited at GenBank under the accession number JAOEGU000000000 (https://www.ncbi.nlm.nih.gov/nuccore/JAOEGU000000000). The raw sequence data (.fastq) have been deposited in the SRA database under the Bioproject PRJNA883059 (https://www.ncbi.nlm.nih.gov/sra/PRJNA883059) and Biosample SAMN30959511.

## Value of the Data

• Draft genome sequence data of *Methylobacter psychrophilus* Z-0021 provide fundamental knowledge on the functional potential of psychrophilic methanotrophs mitigating methane emissions from cold, boreal and arctic terrestrial and aquatic ecosystems, and give insights into their biotechnical applicability.
• This data can be utilized by researchers in biogeochemistry, (environmental) microbiology, biotechnology and circular economy.
• This data can be used in predicting the function of methanotrophs in natural, cold ecosystems under variating physicochemical conditions as well as in developing methane-based bioproduct platforms to be utilized in cold regions.

## 1 Objective

Psychrophilic (i.e., cold-loving) methanotrophic bacteria are widely distributed and play an important role in methane removal in cold, boreal and arctic soil and aquatic ecosystems [1], [2], [3]. They could also be applied in the bioconversion of biogas- and natural gas methane into value-added products in cold environments [4]. Therefore, genomic data of psychrophilic methanotrophs is needed to provide important insights into their functional capabilities. However, psychrophilic methanotroph isolates and consequently their genomes are currently rare [3]. A psychrophilic methanotroph, *Methylobacter psychrophilus* Z-0021, was isolated from tundra soil during the 1990s [5]. Despite being deposited into All-Russian Collection of Microorganisms – VKM (VKM), the isolate was not available as a pure culture at early-mid 2000s when a psychrotrophic, methanotrophic arctic wetland soil isolate, *Methylobacter tundripaludum* SV96 was characterized, thus, restricting a DNA-DNA hybridization comparison between SV96 and Z-0021 isolates [6]. Leibniz Institute, DSMZ-German Collection of Microorganisms and Cell Cultures GmbH (DSMZ) had also received a culture of the strain, but it had been contaminated (personal communication with DSMZ staff). Fortunately, DSMZ could re-purify the strain from their stocks and make it publicly available during 2022 (https://www.dsmz.de/collection/catalogue/details/culture/DSM-9914). We hereby provide the first draft genome of *Methylobacter psychrophilus* Z-0021 (DSM 9914).

## 2 Data Description

**Fig. 1:**
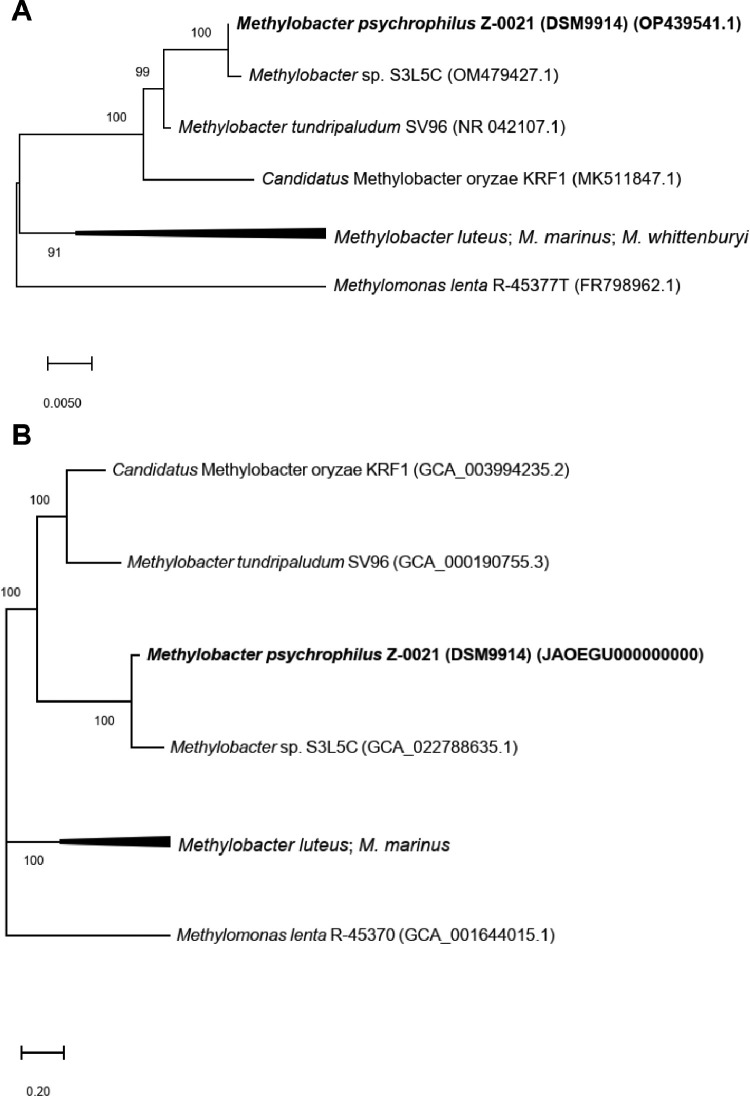
(A) Phylogenetic tree based on 16S rRNA genes and (B) Genome-wide phylogenomic tree based on protein alignments (PhyloPhlAn). Both trees were constructed using the maximum likelihood algorithm with the GTR model for the 16S rRNA gene tree (in A) and PROTCATLG model for the genome-wide tree (in B). Numbers at the nodes indicate the percentage of occurrence in 100 bootstrapped trees. The scale bars indicate the number of substitutions per nucleotide (in A) and amino acid position (in B). *Methylobacter psychrophilus* Z-0021 (DSM 9914) is highlighted in bold.

The full statistics of *de novo* assembly and genome characteristics of strain Z-0021 are reported in Table 1. The draft genome of Z-0021 consisted of 156 contigs, with 4691082 bp in total length, the G+C content of 43.1%, 4074 coding sequences, 4 rRNA and 41 tRNA genes. DSMZ also re-sequenced the 16S rRNA gene of the strain Z-0021 in 2022 (Genbank: OP439541; https://www.ncbi.nlm.nih.gov/nuccore/op439541). The resequenced 16S rRNA gene differed slightly from the originally deposited (NR_025016, deposited in 1999), sharing 99.7% similarity, which could be possibly attributed towards the errors associated with the sequencing in 1990s. In comparison to *Methylobacter* spp., the resequenced Z-0021 16S rRNA gene demonstrated high sequence similarities with the 16S rRNA gene of a recently isolated psychrophilic methanotroph, *Methylobacter* sp. S3L5C (99.7% similarity) [3], and *M. tundripaludum* SV96 (99.3%), while the 16S rRNA gene similarity with other *Methylobacter* strains was much lower (95.4-98.0%) (Fig. 1A). The similarity between 16S rRNA genes of strains S3L5C and SV96 was also relatively high (98.9%) (Fig. 1A). Likewise, in genome-level analyses, the strain Z-0021 showed high similarity towards *Methylobacter* sp. S3L5C genome (Fig. 1B). The Z-0021 strain demonstrated an average nucleotide identity (ANI) of 92.9% and 78.2% and a digital DNA-DNA hybridization (dDDH) value of 50.6% and 22.0% with *Methylobacter* sp. S3L5C and *M. tundripaludum* SV96, respectively. The genomes of other *Methylobacter* strains had lower similarities, i.e 76.3-76.8% ANI and 19.9-20.5% dDDH, compared to the Z-0021 genome (Fig. 1B). The similarity in genomes between strains S3L5C and SV96 was 78.1% ANI and 21.8% dDDH. Despite sharing high 16S rRNA gene identities among strains Z-0021, S3L5C and SV96 (≥98.9%; higher than the 98.65% threshold to delineate unique species [7,8]), their genome-level differences based on ANI (≤92.9%) and dDDH (≤50.6%) were below the 95% and 70% species-level thresholds, respectively [8], [9], [10], [11], [12]. Hence, based on the *in silico* genome analyses, *Methylobacter psychrophilus* Z-0021, *Methylobacter tundripaludum* SV96 and *Methylobacter* sp. S3L5C are different species.

**Table 1:** Statistics of *de novo* genome assembly and genome characteristics of strain *Methylobacter psychrophilus* Z-0021 (DSM 9914)

Feature	Strain Z-0021
Total sequence length (bp)	4691082
Number of contigs	156
N50 (bp)	80242
G + C - content (%):	43.1
Number of coding sequences (CDS)	4074
Number of 5S, 16S and 23S rRNA genes	2 (5S), 1 (16S), 1 (23S)
Number of tRNA genes	41
Number of repeat regions	4

Preliminary gene annotation analyses were done based on the KEGG database. Strain Z-0021 genome contained key genes associated with CH_4_ oxidation including both particulate (pmoCAB) and soluble (mmoXYBZDC) methane monooxygenases, but it did not contain the pxmABC operon. For the conversion of methanol to formaldehyde, the genome contained both calcium- (mxaFJGIACKLD) and lanthanide-dependent (xoxF) methanol dehydrogenases. Genes involved in tetrahydromethanopterin (H4MPT)-mediated pathway, catalyzing the conversion of formaldehyde into formate, were also present in the genome. The genome also contained genes encoding the RuMP pathway [for carbon (formaldehyde) assimilation] and the oxidative TCA cycle. Furthermore, the genome included genes encoding N_2_ fixation (nitrogenase, nifDKH) and assimilation of nitrate (nitrate reductase nasA and nitrite reductase nirBD).

## 3 Experimental Design, Materials and Methods

DSMZ repurified *Methylobacter psychrophilus* Z-0021 (DSM 9914) from their stocks and made it publicly available during 2022 (see details of the strain Z-0021 and its cultivation conditions: https://www.dsmz.de/collection/catalogue/details/culture/DSM-9914).

For this work, the DNA extraction, as well as the genome sequencing and genome assembly of Z-0021 was ordered as a commercial service from DSMZ. Genomic DNA extraction was carried out using MasterPure™ Gram Positive DNA Purification Kit from Epicentre®Biotechnologies Germany according to the manufacturer´s instructions. Sequencing library was prepared using Nextera XT DNA Library Preparation Kit (Illumina®, USA). Library was sequenced on NextSeq 550 Sequencing System using NextSeq 500/550 High Output Kit v2.5 (Illumina®, USA).

The genome was assembled via SpaDES (v. 3.14) on short read genome data [13]. The protein sequences were predicted using Prodigal (v. 2.6) and the contigs were preliminarily annotated using Prokka (v. 1.8) [14,15]. Further annotation of the predicted protein sequences was done based on the KEGG database using KofamKOALA (https://www.genome.jp/tools/kofamkoala/; accessed on 1 Sep 2022) [16]. The genome-wide phylogenetic tree was built from protein alignments generated in PhyloPhlAn (version 3.0.58; PhyloPhlAn database including 400 universal marker genes and “-diversity low” - argument) [17] using the maximum-likelihood algorithm (PROTCATLG − model) with 100 bootstrap replicates in RAxML (version 8.2.12) [18]. Average nucleotide identities with reference genomes were calculated using ANI calculator (http://enve-omics.ce.gatech.edu/ani/, accessed 1 Sep 2022) [9]. Digital DNA-DNA hybridization (dDDH) comparisons with reference genomes were done using the Type Strain Genome Server (TYGS) online service (https://tygs.dsmz.de/; accessed on 1 Sep 2022) [19]. In addition, a 16S rRNA gene-based phylogenetic tree was constructed in Mega X using the maximum-likelihood algorithm (generalized time-reversible model) with 100 bootstraps [20].

## Ethics Statement

This work meets the ethical requirements for publication. Furthermore, this work did not involve human subjects, animal experiments and data collected from social media platforms.

## CRediT Author Statement

**Antti J. Rissanen:** Conceptualization, Investigation, Writing – original draft, Visualization, Project administration, Funding acquisition; **Rahul Mangayil:** Conceptualization, Investigation, Writing – review & editing, Investigation, Funding acquisition; **Mette Marianne Svenning:** Conceptualization, Investigation, Writing – review & editing; **Ramita Khanongnuch:** Conceptualization, Investigation, Writing – review & editing.

## Declaration of Competing Interest

The authors declare that they have no known competing financial interests or personal relationships that could have appeared to influence the work reported in this paper.

## Data Availability

JAOEGU000000000: Methylobacter psychrophilus strain Z-0021, whole genome shotgun sequencing project (Original data) (Genbank).
